# Genome Characterization of the First Mimiviruses of Lineage C Isolated in Brazil

**DOI:** 10.3389/fmicb.2017.02562

**Published:** 2017-12-22

**Authors:** Felipe L. Assis, Ana P. M. Franco-Luiz, Raíssa N. dos Santos, Fabrício S. Campos, Fábio P. Dornas, Paulo V. M. Borato, Ana C. Franco, Jônatas S. Abrahao, Philippe Colson, Bernard La Scola

**Affiliations:** ^1^Laboratório de Vírus, Departamento de Microbiologia, Instituto de Ciências Biológicas, Universidade Federal de Minas Gerais, Belo Horizonte, Brazil; ^2^Departamento de Microbiologia, Imunologia e Parasitologia, Instituto de Ciências Básicas da Saúde, Universidade Federal do Rio Grande do Sul, Porto Alegre, Brazil; ^3^College of Veterinary Medicine and Agronomy, University of Brasília, Brasília, Brazil; ^4^CNRS 7278, IRD 198, INSERM 1095, UM63, IHU – Méditerranée Infection, AP-HM, Unité de Recherche sur les Maladies Infectieuses et Tropicales Emergentes, Aix-Marseille Université, Marseille, France

**Keywords:** *Mimiviridae*, pan-genome, genomics, giant virus, mimivirus

## Abstract

The family *Mimiviridae*, comprised by giant DNA viruses, has been increasingly studied since the isolation of the *Acanthamoeba polyphaga mimivirus* (APMV), in 2003. In this work, we describe the genome analysis of two new mimiviruses, each isolated from a distinct Brazilian environment. Furthermore, for the first time, we are reporting the genomic characterization of mimiviruses of group C in Brazil (Br-mimiC), where a predominance of mimiviruses from group A has been previously reported. The genomes of the Br-mimiC isolates Mimivirus gilmour (MVGM) and Mimivirus golden (MVGD) are composed of double-stranded DNA molecules of ∼1.2 Mb, each encoding more than 1,100 open reading frames. Genome functional annotations highlighted the presence of mimivirus group C hallmark genes, such as the set of seven aminoacyl-tRNA synthetases. However, the set of tRNA encoded by the Br-mimiC was distinct from those of other group C mimiviruses. Differences could also be observed in a genome synteny analysis, which demonstrated the presence of inversions and loci translocations at both extremities of Br-mimiC genomes. Both phylogenetic and phyletic analyses corroborate previous results, undoubtedly grouping the new Brazilian isolates into mimivirus group C. Finally, an updated pan-genome analysis of genus *Mimivirus* was performed including all new genomes available until the present moment. This last analysis showed a slight increase in the number of clusters of orthologous groups of proteins among mimiviruses of group A, with a larger increase after addition of sequences from mimiviruses of groups B and C, as well as a plateau tendency after the inclusion of the last four mimiviruses of group C, including the Br-mimiC isolates. Future prospective studies will help us to understand the genetic diversity among mimiviruses.

## Introduction

Since the serendipitous discovery of *Acanthamoeba polyphaga mimivirus* (APMV) in 2003, dozens of studies have been conducted to describe how widespread and diverse this new viral family is La Scola et al. (2003, 2010), Raoult et al. (2004), Arslan et al. (2011), Colson et al. (2011a,b), Legendre et al. (2012), Boughalmi et al. (2013b,c), Saadi et al. (2013a), Yoosuf et al. (2014a,b). Concomitantly, researchers have been working on the biology and molecular characterization of other mimivirus relatives isolated from several human and environmental samples, the latter of which include cooling water tower, freshwater, saltwater, soil, leech, oyster, and other sources collected in many countries in Oceania, Europe, Asia, Africa, and South America (La Scola et al., 2008; Fischer et al., 2010; Arslan et al., 2011; Yoosuf et al., 2012; Boughalmi et al., 2013a,b,c; Pagnier et al., 2013; Saadi et al., 2013a,b; Campos et al., 2014; Bajrai et al., 2016; Takemura et al., 2016). During those studies, notable sets of genes seemingly encoded by the genome of these new viruses were observed. These included genes encoding tRNA sequences, aminoacyl-tRNA synthetases, and peptide synthesis factors. Equally surprising was that mimiviruses can be associated with small viruses, which were named virophages in analogy to bacteriophages that infect bacterial hosts (La Scola et al., 2008). Some mimiviruses were recently predicted to encode a defense system named the MIMIVIRE, which enables them to target specific virophages (Levasseur et al., 2016). However, all these astonishing discoveries could be the “tip of the iceberg” regarding mimivirus features, as ∼50% of the sequences of these viruses encode proteins that are hypothetical, i.e., without a defined function (La Scola et al., 2003; Raoult et al., 2004).

The mimiviruses have a semi-icosahedral 410–550 nm in diameter capsid, with a symmetry breaking at a single vertex of the particle forming a five-branch star structure, called the ‘stargate’ (Zauberman et al., 2008). The capsid surface is covered, except at the “stargate” vertex, by a 150-nm thick fibril layer, involved in a matrix with a composition initially thought to be similar to peptidoglycan. Although the mimiviruses have been isolated using co-culture on amoebae of the genus *Acanthamoeba*, knowledge about their natural reservoir as well as their host range is still limited. The mimiviruses replicate in the host cytoplasm in a replication factory that is formed after the genome is released (Suzan-Monti et al., 2007; Mutsafi et al., 2010; Colson et al., 2017). The genomes of mimiviruses are comprised by a linear dsDNA molecule that is 0.92–1.22 Mb long and encodes 930–1,178 proteins (Raoult et al., 2004). The genome of the prototype Mimivirus was described to present two inverted repeats of about 900 nucleotides near both extremities, suggesting that the Mimivirus genome might adopt a circular topology during viral replication, as in some other NCLDVs (Raoult et al., 2004).

The family *Mimiviridae* is comprised by two genera, named (1) *Mimivirus*, composed of mimiviruses infecting amoebal species, and (2) *Cafeteriavirus*, a distantly related mimiviruses group comprised by the type species *Cafeteria roenbergensis virus* (CroV; which infects a marine heterotrophic bi-flagellate) International Committee on Taxonomy of Viruses [ICTV], 2017. Other related distant mimiviruses have been associated with CroV, including Organic lake phycodnaviruses and *Phaeocystis globosa* virus (Yutin et al., 2013). The recently described klosneuviruses also seem to be related to *Mimiviridae* members (Schulz et al., 2017). The genus *Mimivirus* can be divided into three lineages A, B, and C, according to phylogenomic data including phylogenies based on conserved core genes, for example family B DNA polymerase and ribonucleotide reductase encoding genes (Boyer et al., 2010; Colson et al., 2012; Legendre et al., 2012; Campos et al., 2014).

We isolated the first Brazilian mimivirus strain, named Samba virus (SBV), from a water sample collected in the Amazon region in 2011. Phylogenomic analyses clustered the SBV into mimivirus lineage A (Campos et al., 2014), which includes the APMV, the prototype species of family *Mimiviridae*. More recently, Brazilian mimivirus strains have been isolated and/or detected from fresh water, oyster, sewage, humans, and both wild and domestic mammals, and their biological and molecular characterization have been reported (Dornas et al., 2014, 2016, 2017; Andrade et al., 2015; Boratto et al., 2015). Curiously, all Brazilian mimivirus strains were classified into mimivirus lineage A, suggesting that this lineage is the most widespread in Brazil (Andrade et al., 2015; Assis et al., 2015; Boratto et al., 2015). In addition, Assis et al. (2015) described the pan-genome of mimivirus lineage A, which was composed of 1129 clusters of orthologous groups (COGs) of proteins encoded by all genomes available at that time. All these data led us to ask more questions about the diversity of mimiviruses circulating in Brazil and resulted in the decision to conduct additional prospective studies. In this way, Dornas et al. (2015), using a panel of protozoa (*Acanthamoeba castellanii* [AC]*, Acanthamoeba polyphaga* [AP]*, Acanthamoeba griffinii* [AG] and *Vermamoeba vermiformis* [VV]), were able to isolate 62 new mimivirus-like strains from sewage, sludge, water, wet soil, and lake sediment collected from different areas of the Pampulha lagoon in Belo Horizonte, Minas Gerais, Brazil (Dornas et al., 2015). A higher prevalence of lineage A mimiviruses (90.3%) was observed, followed by lineage C mimiviruses (6.4%) and lastly lineage B mimiviruses (3.2%). However, neither further analysis of the biological and molecular features of these viruses nor phylogenies were provided, once the classification of these new isolates into lineages was inferred based on BLAST hits obtained against the NCBI nt database.

In this work, we report the molecular and phylogenetic analysis of two Brazilian mimiviruses from lineage C (Br-mimiC): (1) Mimivirus gilmour (MVGM) – isolated from water collected at Pampulha lagoon by Dornas et al. (2015); (2) Mimivirus golden (MVGD) – isolated from golden mussels (*Limnoperna fortunei*) collected from Guaíba Lake, Rio Grande do Sul, Brazil, in July 2014. Both Br-mimiC viruses were isolated using the protozoa AP as support for co-culture. In addition, we conducted an updated pan-genome analysis of all available genomes of mimiviruses from lineages A to C.

## Materials and Methods

### Sample Collection

A collaborative effort involving the Aix-Marseille University (France), and the Federal Universities of Minas Gerais and Rio Grande do Sul (Brazil) was established aiming to conduct prospective studies of giant viruses in different regions and environments in Brazil. All collection procedures were performed with the authorization of IBAMA-SISBIO (number 34293-2). For this work, water samples were collected in sterile tubes from Pampulha lagoon in September 2014, and were directly used for inoculation procedures. In addition, golden mussels (*L. fortunei*) were collected from Guaíba Lake in July 2014 (30°01′59″ S, 51°13′48′ W) (Dos Santos et al., 2016). The mussels were collected from the lake bottom at a depth of 2 m, and they were attached to a metal grid that had been submerged for 6 months before the date of collection. Golden mussels were submerged for 15 min in 70% ethanol for superficial shell decontamination. Subsequently, the valves were opened and the inner water was collected and diluted in 1 mL of saline buffer (PBS). The samples were pooled, totaling eight pools. These pools were homogenized with 1 mL of PBS, centrifuged at 10,000 ×*g*, filtered through a 0.45-μm pore-size membrane. The resulting filtrate was treated with 10 U/μL of Penicillin-GIBCO by Life Technologies to prevent bacterial contamination. All the samples were stored at 4°C until the inoculation procedures.

### Virus Isolation

The MVGM sample was isolated using co-culture of AP strain LINC AP1 previously cultured in a 75-cm^2^ cell culture flask with 30 ml of peptone-yeast extract-glucose medium (PYG) at 30°C for 25 h. The culture supernatant was pelleted by centrifugation, suspended in PAS supplemented with an antibiotic mix containing 10 μL of ciprofloxacin (4 μg/mL; Panpharma, Z.I., Clairay, France), 10 μL of vancomycin (4 μg/mL; Mylan, Saint-Priest, France), 10 μL of colimycin (500 IU/mL; Sanofi Aventis, Paris, France), 10 μL of rifampicin (4 μg/mL; Sanofi Aventis), and 10 μL of fungizone (100 μg/mL; Bristol-Myers Squibb, Rueil-Malmaison, France), and dispensed in 0.5 ml amounts to the wells of a 24-well plate with a suspension cell concentration of 10^6^ cells/ml. After that, 100 μL of samples were inoculated into wells and incubated at 30°C for 4 days. The sub-cultures were performed twice on fresh amoebae in a 1-10th dilution. A negative amoebal control was used in each microplate (Dornas et al., 2015). For the MVGD strain, the amoeba support for co-culture were AP genotype T4 previously cultured in 10 mL of Peptona-Yeast Extract-Glucose (PYG) medium at 30°C in 25-cmł culture flasks supplemented with 50 μg of gentamicin. After 48 h, the cells were harvested and centrifuged. The pellet was re-suspended in sterile PAS (Page’s amoeba saline), and 10^4^ amoebas per well were cultured in 24-well microplates. After 24 h, 100 μL of samples were inoculated into wells and incubated at 30°C for 3 days. The sub-cultures were performed as previously mentioned, and amoeba cells were assessed daily for the presence of viruses and for cytopathic effects on the cell monolayer.

### DNA Extraction and Genome Sequencing

For MVGM strain, viral DNA was extracted with the automated EZ1 Virus Mini-Kit v.2 kit (Qiagen GmbH, Hilden, Germany) according to the manufacturer’s instructions. DNA quality and concentration were checked, using a nanodrop spectrophotometer (Thermo Scientific, Waltham, MA, United States). For the MVGD strain, the supernatant of the *A. polyphaga*-infected cells was collected, and centrifuged at 5,000 ×*g* for 5 min. The cell-free virus particles were pelleted on a 25% sucrose cushion by ultracentrifugation (Sorvall Combi) at 33,000 ×*g* for 2 h at 4°C. The pellet was re-suspended in Tris-EDTA-NaCl buffer (TEN). In order to remove the nucleic acids not protected by the capsid, the preparation was treated with 100 U of DNAse I (Roche) and 100 U of RNAse (Invitrogen) at 37°C for 1 h. Next, the virus DNA was extracted using phenol–chloroform (Sambrook and Russell, 2001) and re-suspended in ultrapure water. The quality and amount of virus DNA was analyzed using a NanoSpec and Qubit apparatus (Life Technologies). Both extracted viral DNA were submitted to sequencing performed in a MiSeq (Illumina) apparatus with paired-end applications (2 bp × 150 bp). The pair-end samples were prepared with a Nextera XT DNA sample prep kit.

### Genome Assembly and Annotation

After sequencing, reads from MVGM and MVGD were *de novo* assembled using Geneious and SPADES softwares. The gene predictions were performed using RAST (Rapid Annotation using Subsystem Technology) (Aziz et al., 2008) and GeneMarkS (Besemer et al., 2001) tools. Transfer RNA (tRNA) sequences were identified using the tRNAscan-SE tool (Schattner et al., 2005). The functional annotations were inferred by BLAST searches against the GenBank NCBI non-redundant protein sequence database (nr) (e-value < 1 × 10^-3^), the set of COGs of the NCLDVs (named NCVOGs) (Altschul et al., 1990; Yutin et al., 2009) and by searching specialized databases through the Blast2GO platform (Conesa et al., 2005). The genome annotations were then manually revised and curated. The predicted open reading frames (ORFs) smaller than 50 amino acids (aa) and that had no hit in any database were discarded. The ORFs longer than 50 aa without hits in any database (ORFans) were kept.

### Comparative Genomic and Pan-genome Analysis

The synteny among mimiviruses from distinct lineages was checked using MAUVE program (Darling et al., 2010). The OrthoMCL tool (Chen et al., 2006) was used to identify the paralog families from Br-mimiC genomes, while Proteinortho5.pl tool (Lechner et al., 2011) was used to identify orthologous gene sequences shared by Br-mimiC. The average amino acid identity (AAI) calculator tool (Rodriguez-R and Konstantinidis, 2014) was used to compare identity between orthologous genes from Br-mimiC strains and representative mimiviruses of lineages A-C. To estimate the size of the pan-genome of the family *Mimiviridae*, their predicted proteins were clustered using the Proteinortho5.pl program (Lechner et al., 2011), using an aa sequence identity of 30% and a sequence coverage of 50% as thresholds. We also described pan-genome and core genes size variation by stepwise inclusion of each new virus annotation in the pairwise comparisons of the gene contents of all available mimivirus genome sequences.

### Phylogeny

The aa sequence alignments and phylogenetic trees were built using the MEGA6 software (Tamura et al., 2013) and the maximum likelihood method. Phylogenetic reconstructions were based on individual alignment of the five core genes, namely the family B DNA polymerase, the D6/D11 helicase, the VV A18 helicase, the D5 primase-helicase, and the Major Capsid Protein. In addition, we performed a hierarchical clustering based on the gene presence/absence pattern of 5443 NCVOGs, using the MeV tool (Eisen et al., 1998) with Pearson correlation as distance metric. The phylogenetic tree was visualized using the FigTree v1.4.3 tool (available online: http://tree.bio.ed.ac.uk/software/figtree/).

## Results

### General Features of Br-mimiC Genomes

The genomes of MVGM (GenBank number: MG602507) and MVGD (GenBank number: MG602508) are double-stranded DNA molecules of 1,258,663 base pairs (bp) (partial sequence) and 1,248,960 base pairs (complete sequence) encoding 1,135 and 1,127 ORFs, respectively. The ORFs length of both Br-mimiC ranged from 37 to 2,907 aa, with an average length of 326 aa. The BLAST analysis (coverage > 90%; identity > 80%; e-value < 10e-5) against the NCBI nr database (updated in October, 2017) identified 1088 and 1090 hits for MVGM and MVGD sequences, respectively. Furthermore, we identified 28 and 19 ORFans into MVGM and MVGD genomes, respectively. In addition, 19 and 18 ORFs without BLAST hit and smaller than 50 aa were not include in the subsequent analysis neither in the final annotation of MVGM and MVGD genomes, respectively.

The comparison between the Br-mimiC viruses genomes showed the presence of 1,042 orthologous proteins, whereas 66 and 61 proteins are unique to MVGM and MVGD, respectively. The set of unique genes of the MVGD included 18 ORFans, besides hypothetical proteins, ankyrins, F-box and FNIP repeat-containing proteins, collagen-like proteins, BTB POZ domain and WD-repeat proteins and a cholinesterase-like protein. With the exception of the cholinesterase-like protein found in the MVGD genome, the set of unique genes of the MVGM was comprised by the same classes of protein, besides a DNA primase and a putative transposase. In addition, the MVGM and MVGD genomes encoded to 551 and 558 proteins without defined function, respectively.

**FIGURE 1:**
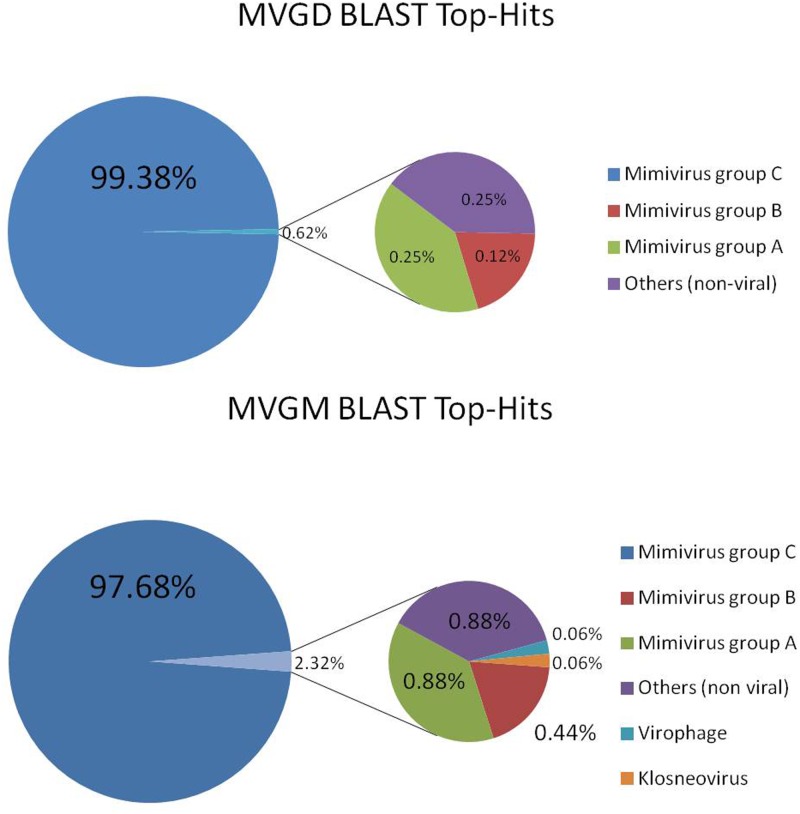
BLAST Top-hit analysis. Graphical distribution of the best BLAST hits for both Br-mimiC gene contents. The analysis was performed using BLASTp algorithm searching with the predicted ORFs from Br-mimiC against the NCBI GenBank non-redundant (nr) protein sequence database using the java-based free software Blast2GO. The mimiviruses of group C were the predominant targets for hits from both Br-mimiC.

**FIGURE 2:**
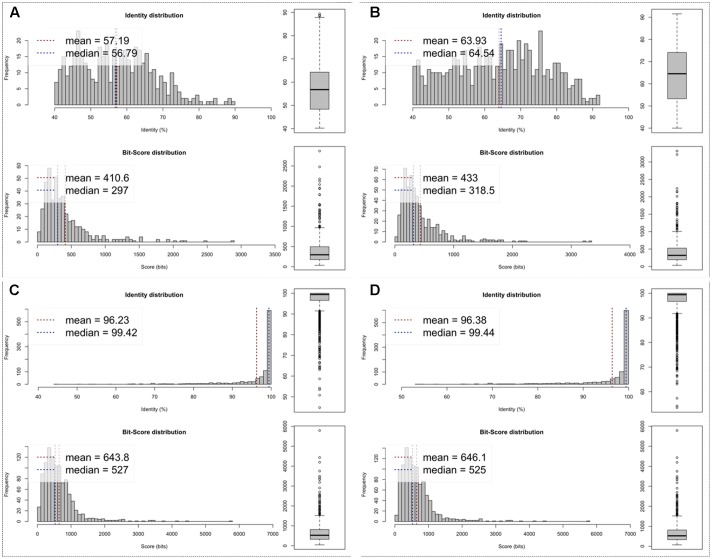
Average amino acid identity (AAI). In this analysis, estimates were reached out using both best hits (one-way AAI) and reciprocal best hits (two-way AAI) between two datasets of proteins from the Br-mimiC isolates and representative strains from mimivírus groups A to C. Plots **(A,B)** demonstrate the AAI between MVGM and mimiviruses from group A to group B, respectively. Plot **(C)** shows the AAI between MVGM and MVGD. Plot **(D)** shows the AAI between MCHV (group C prototype strain) and MVGD.

Both genomes showed a very similar G+C content (∼26%), genome density (∼0.890 genes per kbp), coding percentage (∼88.5%), and average gene length (995 bp). The best hit analysis for the sequences predicted in these Br-mimiC genomes showed the highest percentage (average of 98.5%) of hits against mimivirus group C sequences (**Figure 1**). The average AAI analysis (**Figure 2**) corroborated the best hit analysis showing the greatest AAI value between sequences from Br-mimiC and other mimiviruses of group C (∼96%), followed by mimiviruses of group B (63.9%) and mimiviruses of group A (57.1%). When compared between each other, the Br-mimiC showed an AAI of 96.3% (**Figure 2**). Beyond, the ORFs predicted into Br-mimiC genomes possess orthologs into other mimiviruses, hosts and/or sympatric organisms, beside virophage and other giant viruses. Furthermore, we observed the presence of seven aminoacyl (tyrosyl, cysteinyl, methionyl, arginyl, isoleucyl, asparaginyl, and tryptophanyl) tRNA synthetases (aaRS) in Br-mimiC, which has been described as a signature of mimiviruses of group C (Colson et al., 2013), while mimiviruses of groups A and B encode four and five aaRS, respectively (**Table 1**). Although the best hit analysis has shown a match against a virophage sequence, we did not detect those mimivirus-related virus associated with Br-mimiC.

**Table 1:** Distribution of aminoacyl-tRNA synthetases encoded by mimivirus from group A to group C, besides Br-mimiC isolates.

	Mimivirus strains			
Aminoacyl-tRNA synthetase	Mimivirus A	Mimivirus B	MCHV	Br.mimi C
Tyrosyl	V	V	V	V
Cysteinyl	V	V	V	V
Methionyl	V	V	V	V
Arginyl	V	V	V	V
Isoleucyl	X	V	V	V
Asparaginyl	X	X	V	V
Tryptophanyl	X	X	V	V


*MCHV, Megavirus chilensis; V, presence; X, absence.*

Even sharing several genetic features, such as a low G+C content and large and similar genome sizes and gene repertories, the Br-mimiC isolates presented singular features which allowed distinguishing them as two distinct isolates. One of the main differences between the Br-mimiC viruses is the presence of six tRNA molecules (2x Leu-TAA, Leu-CAA, Trp-CCA, His-GTG, and Cys-GCA) encoded by MGMV, while the MGDV was predicted to encode only three tRNA molecules (Leu-TAA, Leu-CAA, and Trp-CCA). Taken together, these results confirm the isolation of the first mimiviruses of group C in Brazil.

**FIGURE 3:**
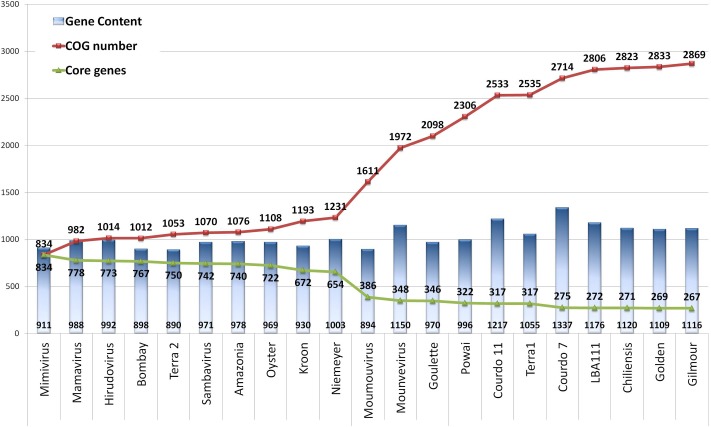
Evolution of the pan-genome (red line) and core genome (green line) size of the family *Mimiviridae.* Numbers at the base of blues bars refer to the number of genes encoded by each virus strain. Numbers at line nodes represent the cumulative COG (red line) and core genes (green line) numbers after the inclusion of a new genome. The COGs definition was performed by using the Proteinortho5 tool, using AAI and coverage of 30 and 50%, respectively.

In order to assess the gene encoding capacity of mimiviruses, we performed an updated pan-genome analysis (**Figure 3**) using all mimivirus genome data available in the NCBI genome database. This analysis will shows us the set of different proteins encoded by all mimiviruses, and will indicate whether the genetic complexity of this group has been fully addressed or not. For this analysis, only complete genome data sets were used, and the result showed a continuous increase in the pan-genome size reaching 2869 COGs, an improvement of 1740 new COGs compared with our previous analysis (Assis et al., 2015) that only considered genomes of mimiviruses of group A. Furthermore, breaks in this rising curve were observed for each new mimivirus representative of the lineages B and C; the number of COGs increased by 380 from lineages A to B, and an additional increase of 208 COGs from lineages A and B to lineage C were observed. In addition, we observed a stabilization trend after the inclusion of the last four mimiviruses of group C, which included the Br-mimiC isolates.

Conversely, we observed a continuous decrease of the core genome after addition of new representatives. An abrupt reduction was only observed after inclusion of the first mimivirus of group B (268 COGs reduction), while a more discrete reduction was observed when sequences from mimiviruses C were included (24 COGs reduction). Further, a stabilization trend of the core genome size was observed for the last five mimiviruses C, including Br-mimiC. In addition, we observed an intra-group divergence of 249 COGs among mimiviruses A, 487 COGs among mimiviruses B, and 563 COGs among mimiviruses C. Altogether, these results highlight a stabilization trend in the pan-genome and core genome evolution of amoeba-associated mimiviruses. In Addition our results showed a great divergence even among viruses from the same group (**Figure 3**).

### Synteny Analysis

**FIGURE 4:**
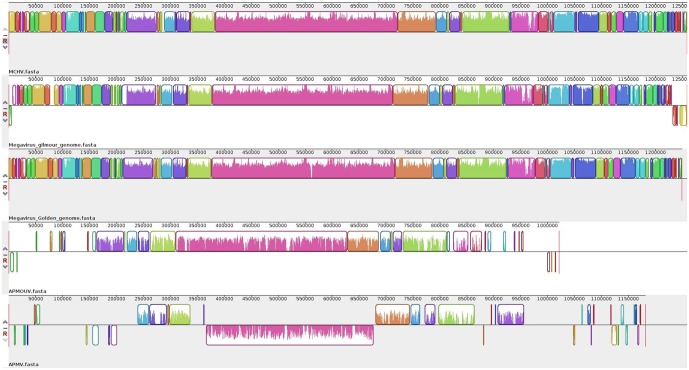
Genome synteny analysis. Schematic genome alignment diagram obtained using the Mauve software package. The analysis was performed using the reference genome of APMV (NC_014649.1), Moumouvirus (NC_020104.1) and MCHV (NC_016072.1), besides the genome sequences of Br-mimiC isolates. The blocks illustrated above X axis are in positive strand (forward sense), while blocks below the X axis are in the negative strand (reverse sense).

The synteny analysis showed very similar genome architectures for *Megavirus chilensis* (MCHV) and the Br-mimiC viruses (**Figure 4**). However, some divergences were observed among mimiviruses C, such as inversions and translocations at both extremities of the MVGM genome, while the MVGD genome better resembled the MCHV genome architecture than that of others. Furthermore, the genome of mimiviruses C showed a better co-linearity with less block brakes in their central region (from ∼250 to ∼950 kb) compared to what is observed at both extremities, which showed an increased number of shorter homologous regions. Curiously, the central region of mimiviruses C genomes showed an overall smaller similarity when compared to the extremities. In addition, these mimiviruses C presented a distinct genome macrosynteny from mimiviruses A and B.

### Phylogeny

**FIGURE 5:**
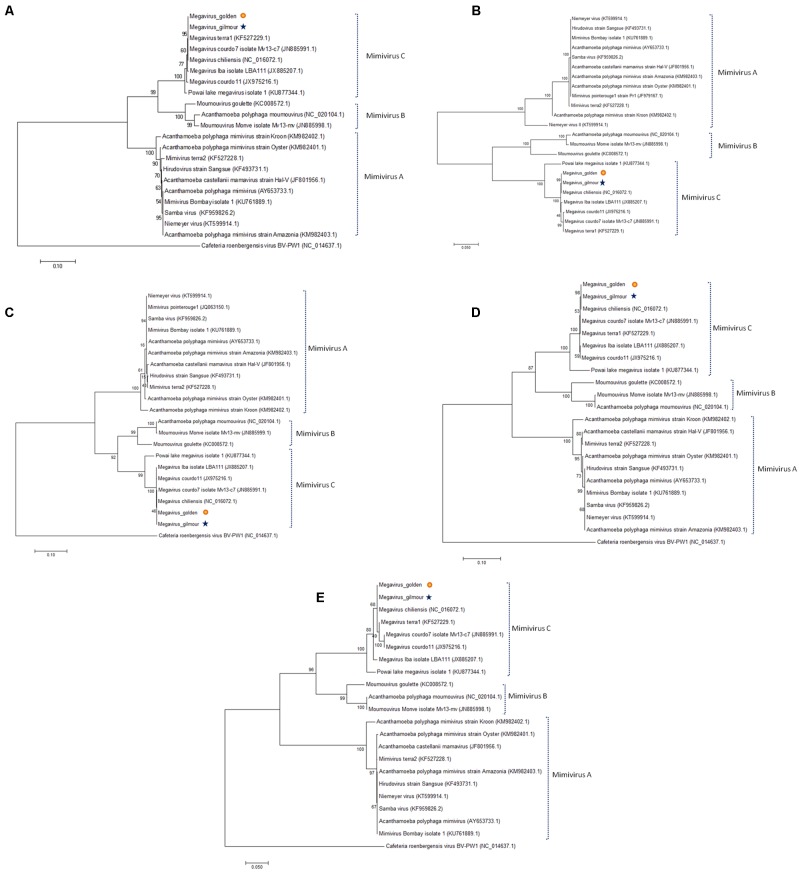
Phylogenetic reconstruction of the NCLDV core genes for different mimiviruses isolates. Here we have done a phylogenetic analysis for many representatives of the different *Mimiviridae* lineages **(A–C)** including for the new Br-mimiC viruses, based on the nucleotide sequences of the five NCLDV core genes, namely the **(A)** major capsid protein, **(B)** the family B DNA polymerase, **(C)** the D6/D11 helicase, the **(D)** VV A18 helicase, and the **(E)** D5 primase-helicase.

**FIGURE 6:**
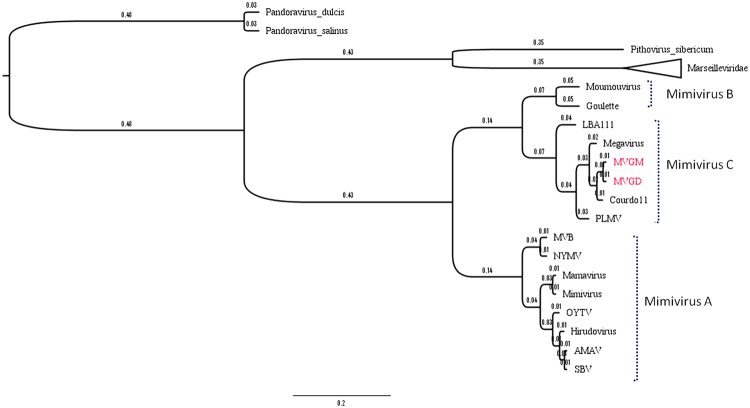
Hierarchical clustering based on the pattern of gene presence/absence for the NCVOGs of different mimivirus isolates. Here we have done a hierarchical clustering tree, based on phyletic patterns, using a presence/absence matrix of 5,443 NCVOG from many representatives of the different *Mimiviridae* lineages (A, B, and C), including the new Br-mimiC viruses, highlighted in red. The distance from horizontal lines connecting the groups represents the time of divergence among the different mimiviruses isolates. The phylogenetic tree was generated using the MeV 4.8.1 tool.

To better understand the evolutionary relationship between the Br-mimiC viruses and other mimiviruses, we performed phylogenetic analyses based on NCLDV core genes including the family B DNA polymerase, the VV A18 helicase, the D5 helicase, the D6/D11 helicase and the major capsid protein (**Figures 5A–E**). Furthermore, a hierarchical clustering tree (**Figure 6**), based on the phyletic patterns, was constructed using a presence-absence matrix of 5,443 NCVOG (clusters of orthologous genes shared by NCLDV). The phylogenetic trees recurrently clustered the Br-mimiC viruses into mimivirus group C, corroborating all previous analyses. The core genes-based trees showed the close relationship of Br-mimiC isolates to the MCHV isolate, the mimivirus of group C whose genome was first described, in 2011, and that was obtained from Chile. However, the phyletic tree, which highlights the gene presence/absence pattern, showed a close relationship of Br-mimiC with Courdo11 virus, isolated in 2010 by inoculating *Acanthamoeba* spp. with freshwater collected from a river of southeastern France.

## Discussion

In this work, we describe the isolation and genome features of the first two isolates of mimivirus group C from Brazil. Recently, we described the isolation of Samba virus, the first representative of family *Mimiviridae* in Brazil, belonging to mimivirus group A (Campos et al., 2014). Subsequently, in Brazil, several mimiviruses and other giant viruses have been isolated in several biomes and a hospital respiratory-isolation facility, and mimivirus has been more recently detected in human sera (Dornas et al., 2014, 2015, 2017; Andrade et al., 2015; Boratto et al., 2015; dos Santos Silva et al., 2015). However, this is the first time that a mimivirus of group C is isolated in this country, which highlights the diversity of giant viruses in Brazil and how widespread these viruses are. Although the former member of mimivirus group C, *Mimivirus chilensis*, has been isolated in Chile, the remaining isolates of this group have frequently been isolated from environmental and clinical samples collected in Asia, Africa, and Europe (Pagnier et al., 2013). Thus, we believe that as new prospective studies are performed, new isolates might be discovered.

Even though they share many molecular features, as well as biological ones (data not shown), the Br-mimiC viruses can be recognized as two distinct isolates. The MVGM isolate has a genome ∼10 kb larger than the MVGD genome and encodes eight more ORFs than MVGD. The unique proteins of both Br-mimiC were mainly located at the extremities of both genomes, which have been described as suitable regions for horizontal gene transfers and duplication events in large and giant viruses, including in mimiviruses (Shackelton and Holmes, 2004; Colson et al., 2011a; Filee, 2015).

Even though the Br-mimiC viruses show ORFans in their genomes, which demonstrate the uniqueness of these isolates, there are notwithstanding many family ORFans present, which means that many genes are shared between mimiviruses but have no homolog in other organisms, and the majority of those genes remains functionally unresolved. Furthermore, we could see a still increasing pan-genome of the family *Mimiviridae* after the addition of Br-mimiC viral genome sequences, suggesting that new genes with unpredictable function are out there, yet to be discovered. In addition, the abrupt break in the trend of the core genome evolution after inclusion of lineage B sequences is in line with the fact that mimiviruses of lineages B and C are more related between each other than they are related to mimiviruses of lineage A, as also shown in the phylogeny reconstructions.

A more conserved synteny could be observed in the central region of all the mimiviruses genomes that were analyzed compared to the remaining part of the genomes. In contrast, the central region of mimiviruses C showed a lower mean similarity. The central region possesses the most ancient set of genes (Shackelton and Holmes, 2004), which have been subjected to long-term selective pressure during mimivirus evolutionary history. In contrast, termini regions of the genome more frequently incorporate new genes, and these recently acquired genes still have a more conserved profile. The phylogenetic analysis strongly corroborate all data presented above, indisputably showing the clustering of the new Br-mimiC isolates into mimivirus group C, closely related to *Megavirus chilensis*, the prototype of this group also isolated in South America. However, the phyletic analysis, which is based on gene presence/absence patterns that at least partially result from losses and gains, showed a better grouping of Br-mimiC viruses with the Courdo 11 virus isolate, which was isolated in 2010 from river water samples in France.

## Conclusion

The discovery of the Br-mimiC viruses contributes to improving the understanding of mimiviral diversity and ubiquity. Nevertheless, the study of giant viruses is still at its beginning. Additional prospective studies must be conducted with the aim of discovering new relatives of these intriguing micro-organisms. Also, this study and others have showed a large number of sequences with unknown function, showing the need of studies focusing in the functional characterization of proteins encoded by the mimiviruses.

## Author Contributions

FA, PB, and AF-L: data collection and pan-genome analyses. FD, AF, RdS, and FC: samples collection and virus isolation. BLS, PC, and JA: study design. All authors wrote the paper and read its last version.

## Conflict of Interest Statement

The authors declare that the research was conducted in the absence of any commercial or financial relationships that could be construed as a potential conflict of interest.
